# Genome Sequence of Mycobacteriophage Phayonce

**DOI:** 10.1128/genomeA.00598-15

**Published:** 2015-06-18

**Authors:** Welkin H. Pope, Emily Jacobetz, Courtney A. Johnson, Brooke L. Kihle, Margaret A. Sobeski, Madison B. Werner, Nancy L. Adkins, Zachary J. Kramer, Matthew T. Montgomery, Sarah R. Grubb, Marcie H. Warner, Charles A. Bowman, Daniel A. Russell, Graham F. Hatfull

**Affiliations:** Department of Biological Sciences, University of Pittsburgh, Pittsburgh, Pennsylvania, USA

## Abstract

Mycobacteriophage Phayonce is a newly isolated phage recovered from a soil sample in Pittsburgh, PA, using *Mycobacterium smegmatis* mc^2^155 as a host. Phayonce’s genome is 49,203 bp long and contains 77 protein-coding genes, 23 of them having predicted functions. Phayonce shares a strong similarity in nucleotide sequence with phages of cluster P.

## GENOME ANNOUNCEMENT

The Science Education Alliance Phage Hunters Advancing Genomics and Evolutionary Science (SEA-PHAGES) is a research program for freshman students that introduces them to bacteriophage diversity and evolutionary processes (1). Several hundred phages of *Mycobacterium smegmatis* mc^2^155 have been isolated and sequenced, and can be grouped into clusters according to their nucleotide sequence similarity and shared gene contents (2). Because *M. smegmatis* is a close relative of *Mycobacterium tuberculosis*, these phages provide insights into mycobacterial genetics and potential applications for the control of human tuberculosis (3).

Mycobacteriophage Phayonce was isolated from a soil sample in Pittsburgh, PA. Following plaque purification, amplification, and DNA isolation, the genome was sequenced using an Illumina MiSeq and 140-bp single end reads. Reads were assembled using Newbler into a major contig with 285-fold coverage, and the virion DNA ends were determined from pile ups in the assembly to have 12 base 3′ extensions with the sequence 5′-CCTGCCCCCCGA. The Phayonce G+C content is 66.7%. Phayonce has a siphoviral morphology with an isometric head approximately 50 nm in diameter and a tail 300 nm long.

Glimmer and GeneMark were used to generate an autoannotation using both heuristic and *M. smegmatis* models, which was then refined by careful manual inspection. A total of 77 open reading frames were identified and no tRNA genes. BLASTn analysis showed that Phayonce has strong nucleotide similarities with phages BigNuz, Donovan, Fishburne, Jebeks, and Purky positioning Phayonce with these other phages in cluster P (4). The closest relative to Phayonce is Purky (phagesdb.org) and these phages share 85% nucleotide identity over a genome span of 74%. Interestingly, there are four unlinked segments of the Phayonce genome that are closely related to the singleton phage Dori (97% nucleotide identity) (4), suggesting relatively recent evolutionary contact between the ancestors of these phages.

Phayonce forms lightly turbid plaques and has genomic features common to temperate phages including an integrase (gene *32*) and an immunity repressor (gene *33*). The organization of these is similar to other phages using an integration-dependent immunity system in which the *attP* integration site is located within the repressor gene (5). Phayonce is predicted to integrate into an *attB* site overlapping a tRNA^thr^ gene (Msmeg_6152) in the *M. smegmatis* genome. The Phayonce lysis system includes putative holin (gp25), lysin A (gp26), and lysin B (gp27) genes, but is mosaic, with each of these three genes having distinct evolutionary histories. Phayonce also encodes a RecE/RecT system (gp47, gp47), a DNA methylase (gp54) with a closely linked possible type II restriction endonuclease (gp51), and a whiB regulator (gp43). The capsid subunit (gp6) is predicted to fold into an HK97-like structure (6), and a ClpP-like prohead protease (gp4) is implicated in virion assembly.

### Nucleotide sequence accession number.

The Phayonce genome sequence was submitted to GenBank under accession no. KR080195.
